# Utility of Supraclavicular Brachial Plexus Block for Anterior Shoulder Dislocation: Could It Be Useful?

**DOI:** 10.5811/westjem.59272

**Published:** 2023-06-30

**Authors:** Michael Shalaby, Melissa Smith, Lam Tran, Robert Farrow

**Affiliations:** Mount Sinai Medical Center Miami Beach, Miami Beach, Florida

Anterior shoulder dislocations (ASD) represent a common and painful orthopedic injury in the emergency department (ED). The management of ASD varies broadly from manual reductions via scapular manipulation with or without pain medication to procedural sedation and anesthesia (PSA), and various regional anesthesia (RA) techniques. The reduction approach often depends on the patient’s subjective pain response, the expected difficulty of reduction, and the physician’s experience with each method. Of the anesthetic options for difficult shoulder reductions, we generally favor RA techniques over PSA. While several RA techniques have been discussed in the literature, one technique that has yet to be analyzed is the supraclavicular brachial plexus nerve block (SBP). We believe there is evidence to suggest that the SBP would serve as an excellent anesthetic option for patients with ASD and significant pain or an expected difficult reduction.

## ANTERIOR SHOULDER DISLOCATION–DIAGNOSIS AND MANAGEMENT

**Figure 1:**
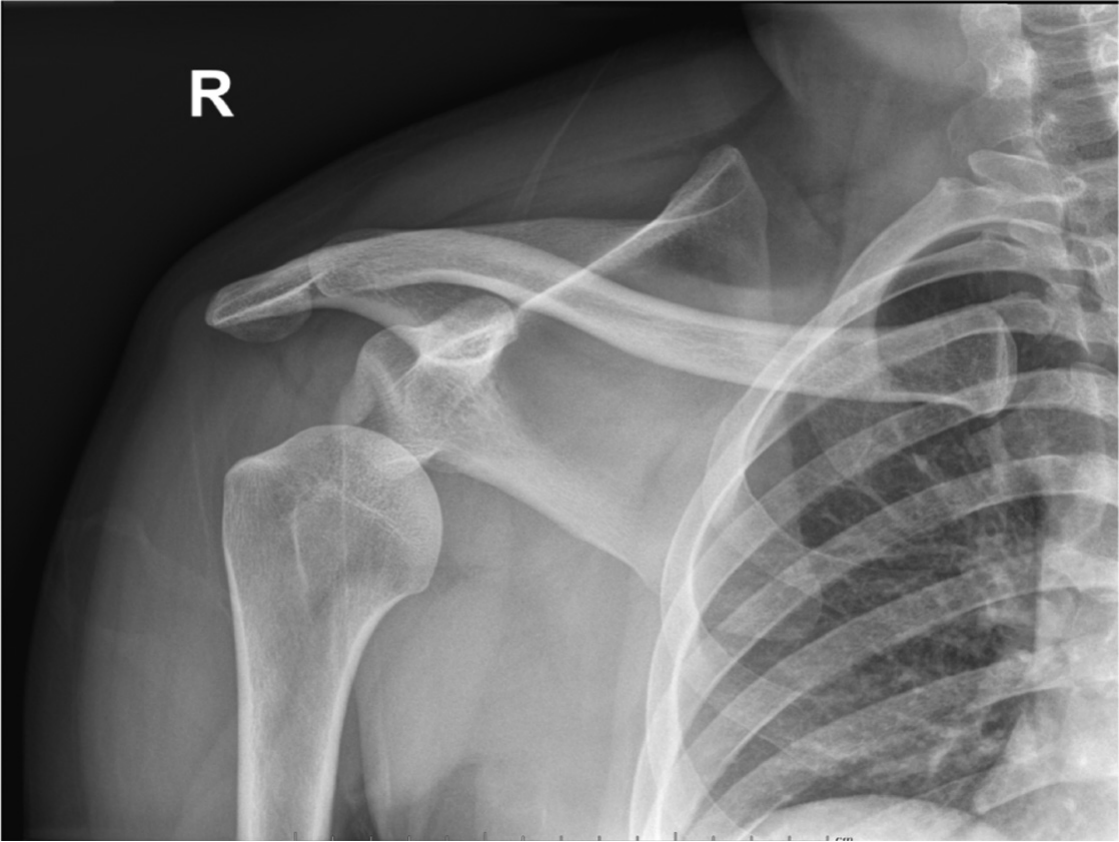
Radiograph of anterior shoulder dislocation, courtesy of Nirav Joshi, MD, Mount Sinai Medical Center, Miami Beach.

**Figure 2:**
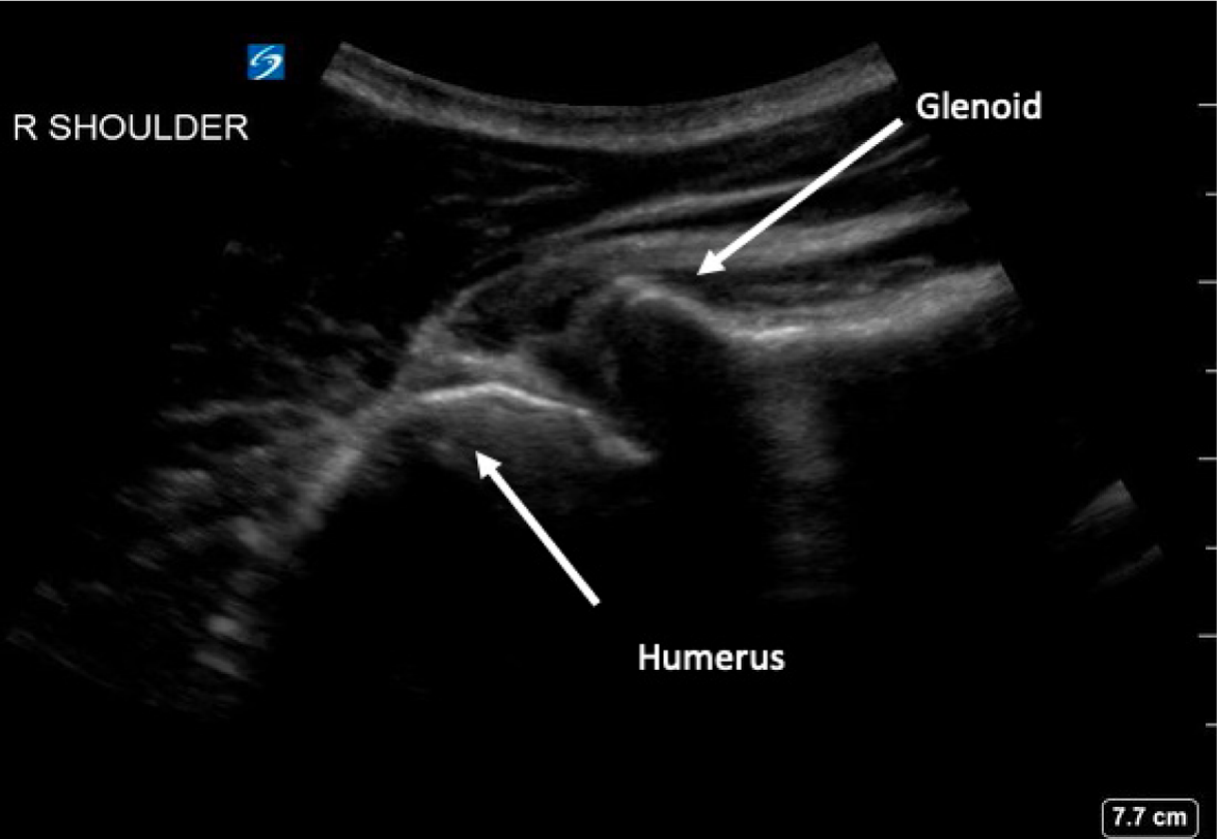
Posterior shoulder ultrasound demonstrating anteriorly dislocated humeral head, courtesy of Michael Rosselli, DO, Mount Sinai Medical Center, Miami Beach.

Anterior shoulder dislocations (ASD) are fairly common in the emergency department (ED); they account for 45% of all joint dislocations and carry a 2% prevalence in the general population.^1^ Because of the significant pain patients often endure, timely management is imperative. Diagnosing ASD is traditionally via radiograph (Figure 1), but emergency physicians are adept at identifying it with point-of-care ultrasound (POCUS) alone with nearly 100% sensitivity and specificity^2^ (Figure 2). Unfortunately, 95% of patients with ASD experience recurrence of this agonizing condition.^3^

Adequate analgesia can be critical to the management of ASD, and failure to obtain first-pass success portends lower rates of closed reduction.^4^ Approaches to reduction include analgesia with intravenous and per os medications, procedural sedation and anesthesia (PSA), and regional anesthesia (RA). The first option, though, generally only blunts pain and thus portends a 16% failure in reduction;^5^ so, we seldom employ it except for patients who are in only mild distress, such as those with recurrent ASD. For the management of most ASD in the ED, the fundamental choice lies between PSA and RA.

To its merit, PSA is analgesic, anxiolytic, anesthetic, and importantly, amnestic; with appropriate dosages patients achieve adequate (although temporary) pain control and do not recall the reduction. Furthermore, PSA allows physicians with little or no experience in RA or other anesthetic procedures (such as intra-articular injection) to reduce ASD successfully. However, PSA is time- and labor-intensive, mandating the presence of a nurse in the room during the entire procedure. Use of PSA also necessitates close patient airway and cardiac monitoring due to the risks of respiratory depression, hypotension, and vomiting.^6^ It generally lengthens ED visits, as patients must reach clinical sobriety before being discharged. Depending on the doses of anesthetics used, patients may take hours to metabolize the induction agents sufficiently.^6^ Emergency physicians also should not overlook the social aspect of PSA, as intoxicated patients often become disinhibited and may feel embarrassed afterward. Lastly, PSA exposes patients to opioids, and 80% of heroin addiction begins with medically dispensed opioids.^7^

## REGIONAL ANESTHESIA—CURRENT USES AND NEW TECHNOLOGIES

Regional anesthesia, on the other hand, circumvents most of the drawbacks of PSA. Regional anesthesia requires only that the performing physician be present in the room with the patient on the cardiac and airway monitor. Patients can remain awake, alert, and oriented, and if performed properly RA can completely anesthetize the affected area, as is the case in brachial plexus (BP) blockade for upper extremity injuries.^8^ Furthermore, depending on the anesthetic used, patients can be discharged home within hours to days of anesthesia post-reduction, an especially desirable outcome for patients with fracture-dislocations. Use of RA can also decrease the length of stay^9^ and increase patient satisfaction.^10^

Should an attempted nerve block either provide subpar anesthesia or fail completely, the administration of local anesthetics does not preclude the subsequent use of PSA. However, like most invasive interventions RA poses its own perils, including risk of nerve damage, vascular puncture, pneumothorax (depending on anatomic location) and, most importantly, local anesthetic systemic toxicity, thus necessitating cardiac and airway monitoring.^11^ Yet unlike with PSA, steps can be taken to avert such complications, which are most often minimized with increasing physician skill and are not the result of patient physiology alone. There are many RA options for emergency physicians to reduce ASD: interscalene and infraclavicular BP blockade, suprascapular nerve block, and intra-articular injection of local anesthetic into the glenohumeral joint.^6^ Most recently, Yu et al published a case series on the successful use of the retroclavicular approach to the infraclavicular region (RAPTIR), an infraclavicular BP block, for anterior shoulder reduction.^6^

The use of RA in the ED is endorsed by the American College of Emergency Physicians (ACEP), which in a 2021 policy statement emphasized that ultrasound-guided nerve blocks are “not only within the scope of the practice of emergency physicians, but represent a core component of a multimodal pathway to control pain for patients in the emergency department.”^12^ Academic emergency medicine leaders are on board with ACEP’s statement: In a survey study of program directors and ultrasound department directors, nearly all agreed that RA is an integral part of resident education.^13^ Curricula for teaching RA are being actively developed across residency programs,^14^ and residents continue to show an eagerness to learn and the capacity to perform RA techniques.^15^ When it is clinically appropriate, we favor RA in the ED for the reduction of fractures and dislocations.

### Supraclavicular Brachial Plexus Block—Why It Will Work for Anterior Shoulder Dislocation

**Figure 3:**
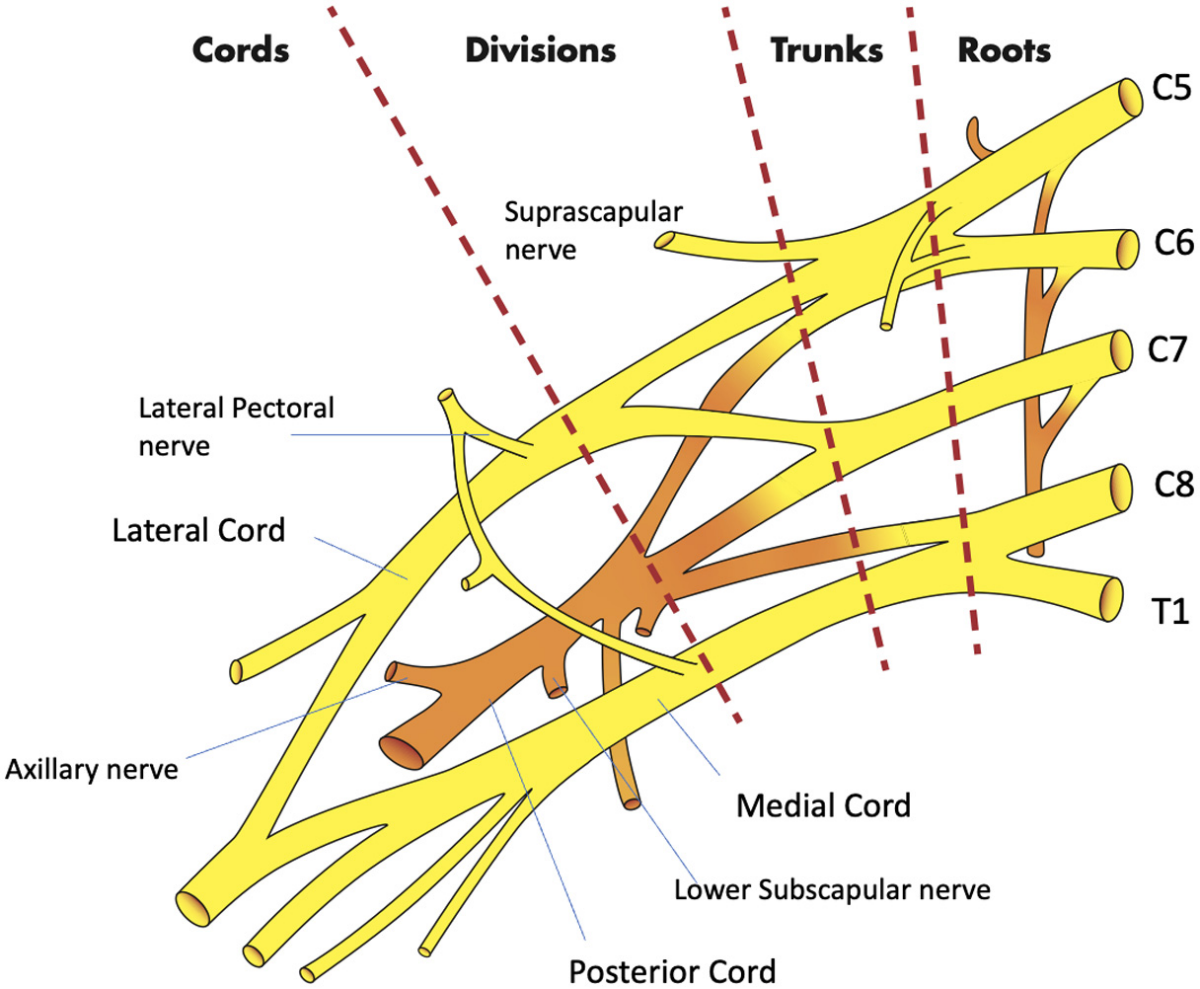
The brachial plexus. Image courtesy of Anthony Casazza.

**Figure 4:**
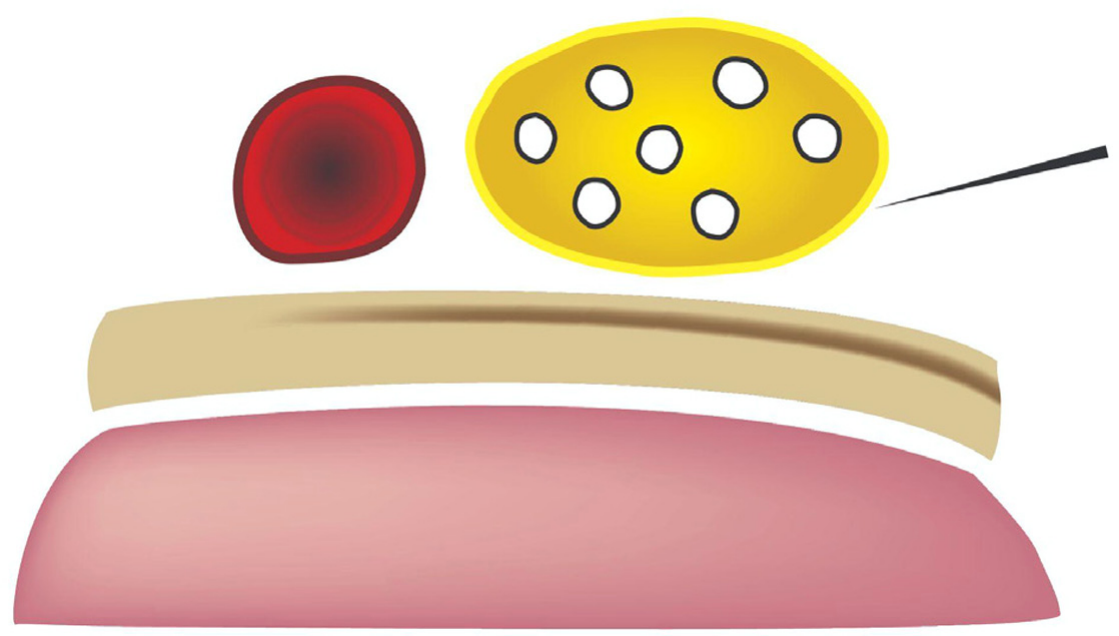
Supraclavicular brachial plexus (SBP) (yellow), subclavian artery (red), first rib (beige), and pleura (pink). The needle (upper right corner of the image) approaches the SBP from lateral to medial. Image courtesy of Anthony Casazza.

The SBP block anesthetizes the upper limb and shoulder by targeting all trunks and divisions of the BP^16^ (Figure 3). The SBP block can be performed in any position in which the patient is comfortable, as long as the patient’s head is turned to the contralateral side. After placing a high-frequency (10–5 megahertz) linear probe immediately superior to the clavicle, the SBP is visualized with the subclavian artery medial, the first rib caudal, and the pleura deep to the rib^16^ (Figure 4). Many features of the SBP block make its application in the ED favorable for both physician and patient. First, the SBP is very shallow (usually 1–2 centimeters deep to the skin) and easily identified when all other necessary structures (subclavian artery, first rib, pleura) are also visible within the field of view on POCUS. Moreover, the first rib acts as a backstop, so that should the physician overshoot the SBP with the block needle, a resulting pneumothorax may be avoided.

Additionally, the setup is simple: depending on the patient’s body habitus the SBP block only requires a 22 gauge needle and usually not a spinal needle, as well as a short linear probe easily held and manipulated in one hand. If the physician is skilled enough, she or he can directly aspirate and inject with a syringe instead of having an assistant control aspiration and injection with a syringe connected to the needle via tubing. We prefer the Miller weight-based dosing for local anesthetic, based on the patient’s calculated ideal body weight.^17^ However, in our experience, usually 15 milliliters of local anesthetic injected directly into the plexus sheath is adequate for anesthesia of the upper limb, which frequently entails less than the maximum amount of local anesthetic for an adult patient.

**Figure 5:**
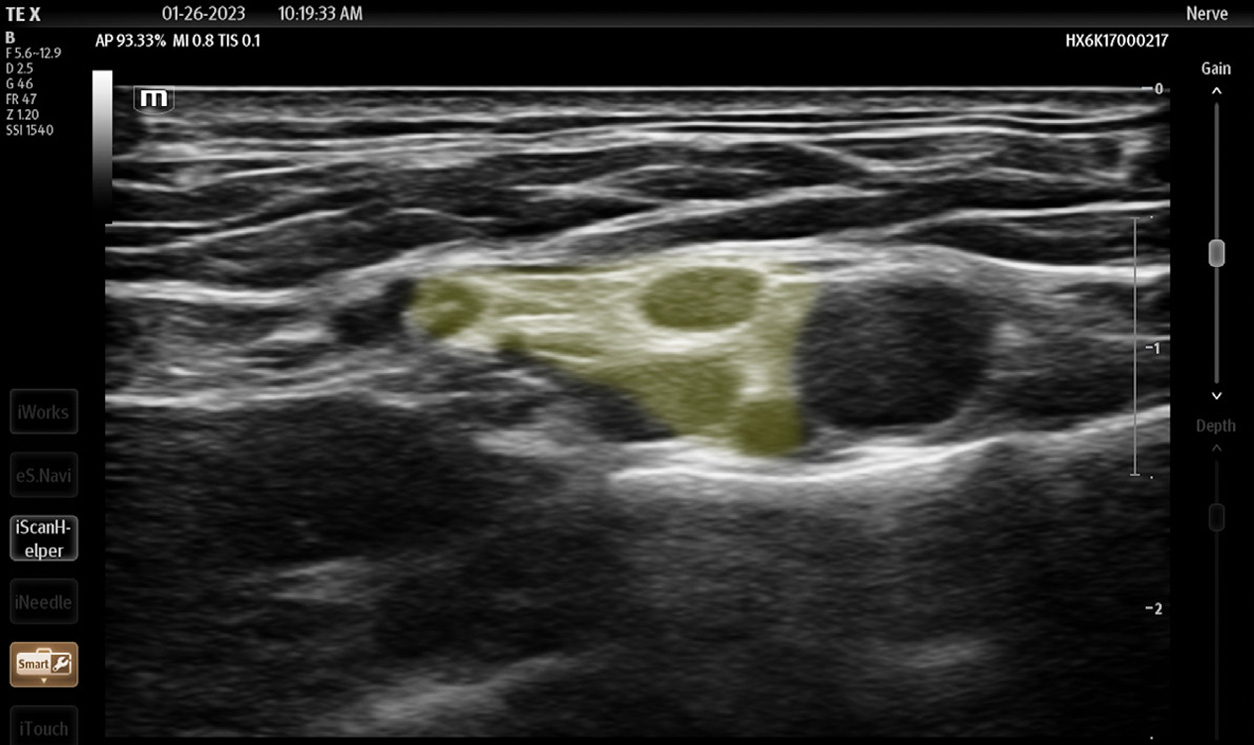
Ultrasound image of supraclavicular brachial plexus highlighted in yellow (Smart Nerve), Mindray TE X, Mindray North America, Image courtesy of Andrew Parrish.

**Figure 6:**
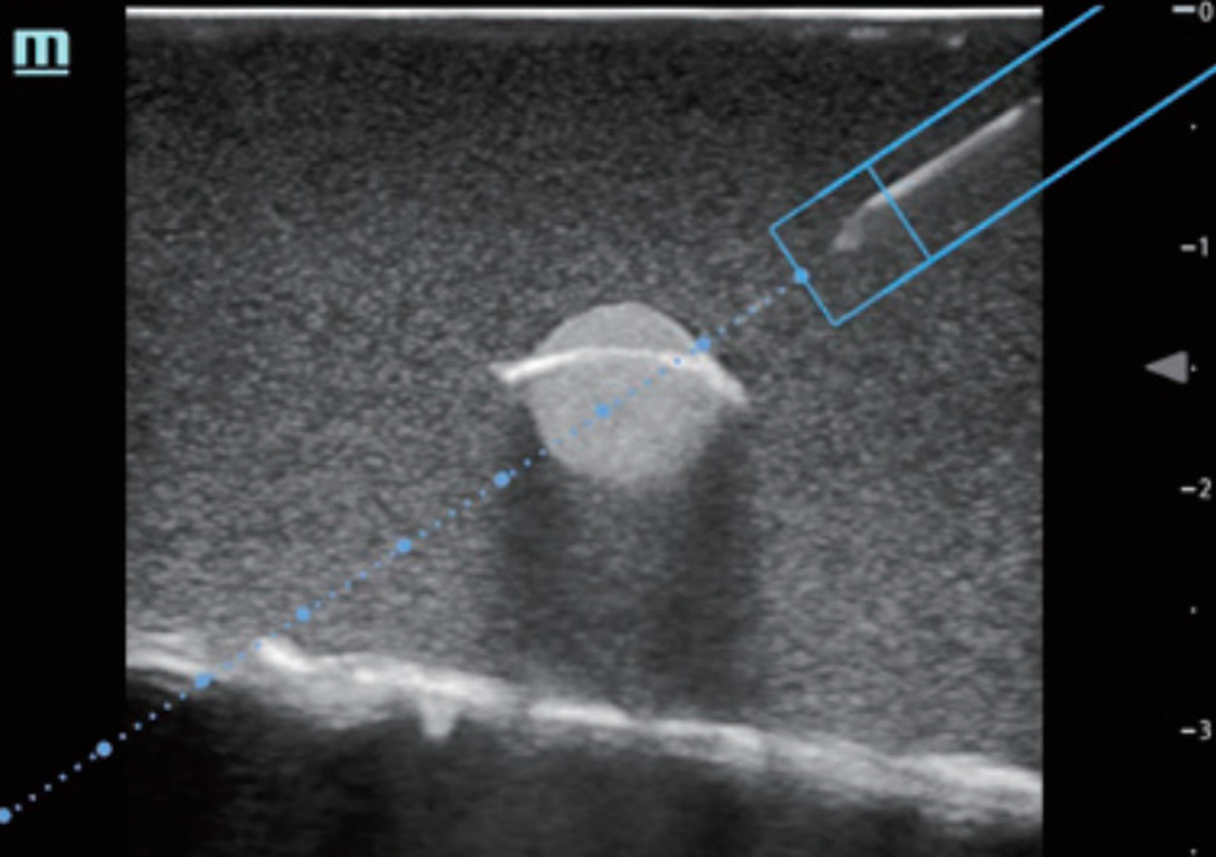
Magnetized needle trajectory guidance (ESpacial Navi), Mindray TE7, Mindray North America.

Although the SBP block has classically been used for more distal procedures,^16^ the spread of anesthetic within the BP sheath after an SBP block supports its use for the reduction of ASD. The glenohumeral joint and intrinsic shoulder muscles derive their sensory innervation from the suprascapular, lateral pectoral, axillary, and lower subscapular nerves.^18^ By anesthetizing these sensory nerves, the SBP block anesthetizes the glenohumeral joint capsule and alleviates pain induced by an ASD. Additionally, disruption of the other cords of the BP weakens or paralyzes muscles of the upper extremity that actively resist reduction, even in patients who are under the influence of PSA.^6^ For patients who suffer from associated Hill-Sachs deformity, which induces lingering discomfort post-reduction, longer acting anesthetics such as bupivacaine or ropivacaine provide hours or days of relief.^19^ And while the SBP is already easy to perform, newer cart-based POCUS systems are equipped with settings that highlight the BP and provide needle trajectory guidance (TE X, Mindray North America, Mahwah, NJ) (Figures 5, 6). The SBP block is comfortable for patient; block performance allows patients to remain in their preferred position on the stretcher.

Of note, although the SBP block carries a low complication rate, some experts are wary of intracluster injection. Gadsen et al argue that based on cadaveric studies, the inadvertent, sub-perineural spread of anesthetic may result in axonal damage.^20^ Other complications include pneumothorax, hemidiaphragmatic paralysis, and subclavian artery puncture, due to the proximity of both these structures to the SBP. Clinically, however, the SBP is safe. Ultrasound use for the SBP block has been demonstrated to nearly eliminate the risk of pneumothorax and neuronal injury.^21^–^24^ Furthermore, in our opinion if the treating physician maintains appropriate needle visualization throughout the procedure—which should be feasible given the shallow nature of the SBP block—both the lungs and the subclavian artery will remain well out of the needle trajectory. And while most often the performance of an SBP block will require two operators (one to inject and one to guide the needle), possibly limiting its use in settings with limited staffing, a skilled physician can perform the entire procedure with a single needle and syringe. Overall, the SBP block is safe, convenient, comfortable, and suitable for ASD.

The interscalene block, commonly used for shoulder pathology in the ED, also provides excellent anesthesia for the shoulder by targeting the C5 to C7 nerve roots.^25^ However, in our opinion, it is more difficult to perform: The target nerves are smaller, harder to visualize on POCUS, and probe manipulation is more technically challenging because the BP at the level of the anterior and middle scalene muscles is farther up the neck and requires a steadier grip on the probe, as opposed to the more favorable position of the probe resting on the clavicle in the SBP block. In a patient in severe distress who has trouble lying still, the performance of this block may represent a difficult task. Lastly, the interscalene block is known to carry a slightly higher risk of permanent nerve damage.^26^ Intra-articular injection, on the other hand, anesthetizes the glenohumeral joint capsule but does not paralyze spastic muscles and, therefore, it does not make ASD reduction easier for the patient or the physician.

## DISCUSSION

Anterior shoulder dislocation is prevalent, painful, and tends to recur in patients with previous episodes. Reduction without sufficient analgesia may be intolerable for patients,^5^ and failed initial attempts lead to more open reductions.^4^ Thus, all efforts should be made to make patients comfortable prior to reduction. Although most physicians are familiar with PSA, it is time-consuming, labor-intensive, increases the length of stay for patients in the ED, risks airway compromise, exposes patients to opioids, and does not impart lasting analgesia. On the other hand, RA is learnable, involves fewer personnel, and generally does not pose airway risk or oversedation. From our experience, patients leave the ED satisfied, in a timely manner, and with potentially lasting pain relief.

It should also be noted that we practice in a community-academic setting and often work shifts without resident physicians to assist with performing RA. Newer ultrasound systems make RA more user-friendly and less intimidating for physicians. The SBP block provides reliable anesthesia, is easy to perform, safe, and allows patients to remain in a comfortable position. Moreover, the use of the SBP block is already commonplace prior to shoulder surgery.^27^ While the interscalene block is also effective, in the setting of an ASD it may be much more technically difficult for the operator. Intra-articular injection is also helpful for pain but does not paralyze muscles. Therefore, we propose that a non-inferiority trial be undertaken to investigate the feasibility and effectiveness of the SBP block compared to the interscalene block or intra-articular injection for the reduction of ASD in the ED.

## CONCLUSION

The supraclavicular brachial plexus nerve block should provide anesthesia for patients with an anterior shoulder dislocation by targeting all sensory nerves responsible for nociception associated with an ASD, as well as weakening/ paralyzing muscles that actively resist reduction due to spasms. If an infraclavicular BP block provides anesthesia for ASD,^6^ why should a SBP block not as well?
